# Cryo-EM structures reveal intricate Fe-S cluster arrangement and charging in *Rhodobacter capsulatus* formate dehydrogenase

**DOI:** 10.1038/s41467-020-15614-0

**Published:** 2020-04-20

**Authors:** Christin Radon, Gerd Mittelstädt, Benjamin R. Duffus, Jörg Bürger, Tobias Hartmann, Thorsten Mielke, Christian Teutloff, Silke Leimkühler, Petra Wendler

**Affiliations:** 1 Institute of Biochemistry and Biology, Department of Biochemistry, University of Potsdam, Karl-Liebknecht Strasse 24-25, 14476 Potsdam-Golm, Germany; 2 Institute of Biochemistry and Biology, Department of Molecular Enzymology, University of Potsdam, Karl-Liebknecht Strasse 24-25, 14476 Potsdam-Golm, Germany; 3 Max-Planck Institute of Molecular Genetics, Ihnestrasse 63-73, 14195 Berlin, Germany; 4 Charité, Institut für Medizinische Physik und Biophysik, Charitéplatz 1, 10117 Berlin, Germany; 5 Department of Physics, Freie Universität Berlin, Arnimallee 14, 14195 Berlin, Germany; 6 Present Address: Ferrier Research Institute, Victoria University of Wellington, Kelburn Parade, Wellington, 6012 New Zealand

**Keywords:** Biocatalysis, Enzyme mechanisms, Cryoelectron microscopy

## Abstract

Metal-containing formate dehydrogenases (FDH) catalyse the reversible oxidation of formate to carbon dioxide at their molybdenum or tungsten active site. They display a diverse subunit and cofactor composition, but structural information on these enzymes is limited. Here we report the cryo-electron microscopic structures of the soluble *Rhodobacter capsulatus* FDH (*Rc*FDH) as isolated and in the presence of reduced nicotinamide adenine dinucleotide (NADH). *Rc*FDH assembles into a 360 kDa dimer of heterotetramers revealing a putative interconnection of electron pathway chains. In the presence of NADH, the *Rc*FDH structure shows charging of cofactors, indicative of an increased electron load.

## Abstract (web summary)

*Rhodobacter capsulatus* NAD^+^ dependent formate dehydrogenase (*Rc*FDH) is a molybdoenzyme that catalyses the reversible oxidation of formate to carbon dioxide, and is of interest for biotechnological applications. Here the authors present the cryo-EM structures of *Rc*FDH as isolated from *R. capsulatus* and in the reduced state with bound NADH, and discuss the enzyme mechanism.

## Introduction

Nature provides a wealth of enzymes that link the two half-reactions of a redox reaction through an electron transfer pathway. Biological processes, such as respiration, anaerobic metabolism and nitrogen fixation, depend on these enzymes and offer inspirational alternatives for difficult chemical conversions. One of these oxidoreductases, the metal-containing formate dehydrogenase, catalyses the following redox reaction:

1 $${\mathrm{CO}}_2 + 2\,{\mathrm{e}}^ - + {\mathrm{H}}^ + \rightleftharpoons {\mathrm{HCOO}}^ - ,\,{\mathrm{E}}^{\circ^{\hbox{,}}} = - 420\,{\mathrm{mV}}$$

In standard state, the equilibrium of the reaction favours formate oxidation at the active site Mo or W atom, which is energetically coupled to the reduction of oxidised nicotinamide adenine dinucleotide (NAD^+^) in cytoplasmic FDHs e.g. from *Rhodobacter capsulatus* and *Cupriavidus necator*. In the presence of an excess of reducing equivalents and under physiological, cellular conditions the reaction is reversible, as shown for several FDHs and formyl-methanofuran dehydrogenases^1–8^. This family of enzymes has also been termed CO_2_ reductases^9^. Enzymatic reduction of carbon dioxide to formate would allow for storage of hydrogen as a fuel for industrial applications^2,9,10^ as well as carbon sequestration from the atmosphere^11,12^, making these enzymes interesting targets for biotechnological applications.

Bacterial FDHs can be divided into different classes, which are distinguished by their subcellular localisation, subunit organisation and cofactor composition^13^. They all share a bis-metal-binding pterin (molybdenum or tungsten) guanine dinucleotide (bis-MGD) cofactor-containing subunit that also binds a proximal [4Fe-4S] cluster, an arrangement that is conserved in other enzymes, e.g. bacterial nitrate reductases^14^. The majority of mesophilic, prokaryotic FDHs coordinate Mo as active site metal. This Mo atom is ligated to two pterin dithiolenes, a sulphur atom and either a cysteine or a selenocysteine. FDHs containing the latter ligand were found to be rather oxygen sensitive^13,15^. All FDHs contain two additional highly conserved residues in the active site, a histidine and an arginine^16,17^. So far, no structural information is available on cytoplasmic, NAD^+^-dependent formate dehydrogenases.

Here, we present the 3.3 Å cryo-EM structures of *Rc*FDH as isolated and in the presence of NADH. The structures reveal a complex arrangement of Fe-S clusters in the dimer, a conserved binding mode of the FdsD to the FdsA subunit and that NADH reduction leads to charging of electron carrying cofactors of *Rc*FDH.

## Results

### Domain architecture of *Rc*FDH

We expressed the *Rc*FDH operon *fdsGBACD* homologously in *R. capsulatus* cells and purified the complex aerobically in the presence of 10 mM azide, a potent inhibitor of FDH (Supplementary Fig. 1a)^18^. The purified complex consists of four subunits, FdsA, FdsB, FdsG and FdsD (Supplementary Figure 1b). In contrast to recent reports on the heterologously expressed *R. capsulatus* enzyme and the FDH from *C. necator*, FdsD is retained as a subunit in the active enzyme (Supplementary Fig. 1c)^6,16,19^. Earlier analyses also support a FdsABGD complex arrangement^20,21^, indicating that likely all NAD^+^-dependent FDHs have a similar overall subunit composition. The homologously expressed *R. capsulatus* enzyme has a similar cofactor saturation and overall activity as compared to the previously reported enzyme expressed in *Escherichia coli*^6^.

To solve the structure of the functional *Rc*FDH complex, we subjected the as isolated, azide inhibited sample to cryo-electron microscopy (cryo-EM) and single particle analysis (Supplementary Fig. 2). Two-fold symmetry of the particle can distinctively be derived from reference-free 2D class averages. Subsequently, we generated a 3D reconstruction with imposed C2 symmetry yielding a final reconstruction with an overall resolution of 3.3 Å as determined by gold-standard Fourier shell correlation (FSC) at 0.143.

**Fig. 1:**
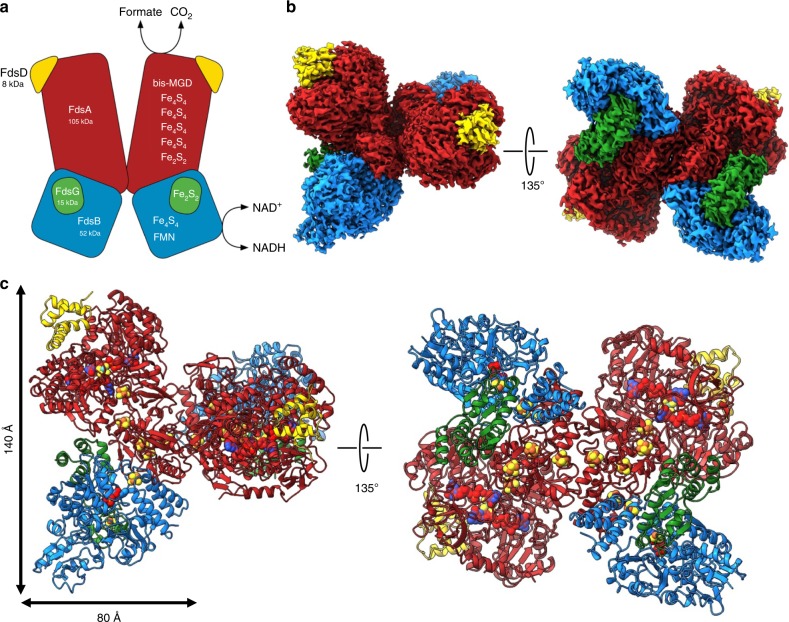
Structural analysis of *Rc*FDH. **a** Schematic representation of RcFDH subunits. Cofactors are indicated. FdsA, red; FdsB, blue; FdsG, green; FdsD, yellow. **b** Three-dimensional reconstruction of C2 symmetric *Rc*FDH. The color code is as in **a**. **c** Ribbon representation of the atomic model derived from the EM structure. Cofactors are shown as spheres.

The *Rc*FDH complex forms a 360 kDa dimer of FdsABGD heterotetramers (Fig. 1). The heterotetramers adopt an elongated structure with dimensions of 140 ×80 ×77 Å and are arranged in an almost perpendicular back-to-back orientation in the dimer. The dimer interface comprises an area of 1369 Å^2^ and is solely formed by the two FdsA (105 kDa, 958 amino acids) subunits. The diaphorase unit consisting of FdsB (55 kDa, 500 amino acids) and FdsG (19 kDa, 150 amino acids) attaches to the N-terminal end of FdsA, whereas FdsD (7 kDa, 71 amino acids) is located near the FdsA C-terminus. The inter subunit contacts FdsA-FdsB, FdsA-FdsD, FdsA-FdsG and FdsB-FdsG are formed with areas of 1436, 1149, 490 and 1754 Å^2^, respectively.

**Fig. 2:**
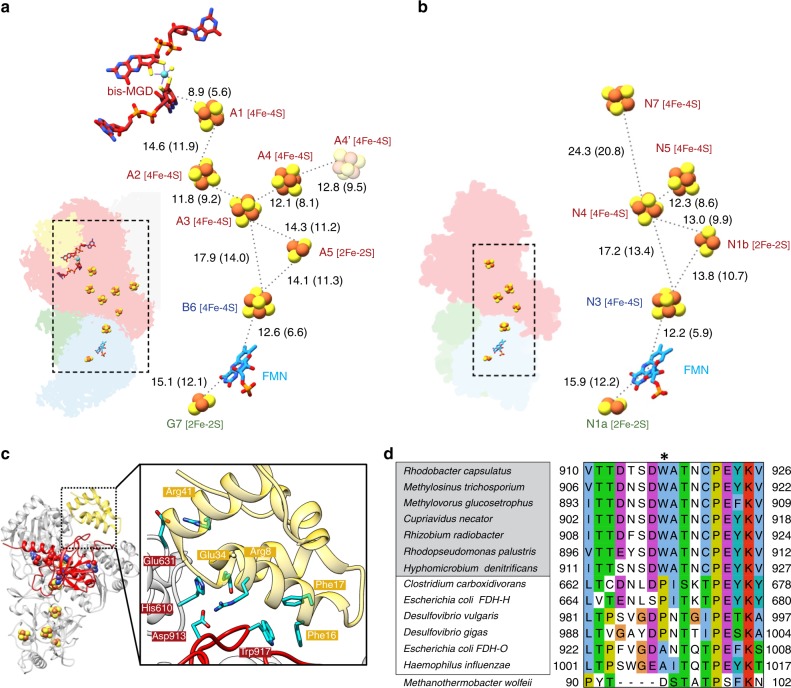
The electron transfer pathway in *Rc*FDH. **a** Overall layout and location of cofactors in *Rc*FDH. The distances between redox centres are given in angstroms, for both centre-to-centre and edge-to-edge (shown in parentheses) measurements. Fe-S clusters are named according to their position in the electron transfer chain and the *Rc*FDH subunit they are located in. The inset depicts the location of the cofactor in the atomic model of *Rc*FDH. **b** Layout and location of cofactors in subunits Nqo1-3 of the hydrophilic domain of *Tt*RC I (PDB-ID: 3iam [10.2210/pdb3IAM/pdb]) depicted in the same orientation as *Rc*FDH in (a). Colour code is as follows I: Nqo1, red; Nqo2, blue; Nqo3, green. **c** The FdsA C-terminus (red) forms a cap domain interacting with proximal and distal MGD as well as FdsD (yellow). Cofactors are shown as spheres. Important interacting amino acids in FdsA and FdsD are indicated and shown in stick representation. **d** Multiple sequence alignment of extended loop in FdsA homologues from organisms with predicted FdsD (grey-shaded) or without predicted FdsD (white-shaded). In *Methanothermobacter wolfeii* the C-terminus of FdsA is replaced by an additional subunit with low homology in the extended loop. The star indicates the position of the conserved Trp residue in NAD^+^ dependent FDHs.

Subunit FdsA binds the bis-MGD cofactor, which coordinates the Mo atom in the active site together with the conserved Cys386 residue. It furthermore contains four [4Fe-4S] clusters and one [2Fe-2S] cluster (Fig. 2a). Three [4Fe-4S] clusters (A2-A4) and the [2Fe-2S] cluster (A5) are located in the N-terminal 250 amino acid FdsA peptide, which shows high structural and sequence similarity to the HoxU subunit of NAD^+^-reducing [NiFe] hydrogenase from *Hydrogenophilus thermoluteolus*^22^ (Supplementary Table 1). As predicted from sequence analysis^14^, the structure of the remaining 700 amino acids of FdsA is most homologous to formate dehydrogenase H (FdhF) of the *E. coli* formate hydrogen lyase complex^15^ and periplasmic nitrate reductase from *C. necator*^23^. Lower homology is found to the tungsten containing FDH from of *Desulfovibrio gigas*^24^ and FDH-N from *E. coli*^25^. The overall structure of FdsA is also highly similar to Nqo3 of respiratory complex I from *Thermus thermophilus* (*Tt*RC I) as predicted by Sazanov and Hinchliffe^26^.

FdsB can be divided into two domains. The N-terminal 100 amino acids form a ferredoxin-like domain highly similar to thioredoxin-like [2Fe-2S] ferredoxin from *Aquifex aeolicus* (Supplementary Table 1)^27^, albeit FdsB lacks the ability to bind a [2Fe-2S] cluster. The second domain, which shows high similarity to Nqo1 of *Tt*RC I^26^, harbours the conserved binding site for NAD^+^, the flavin mononucleotide (FMN) cofactor, and one [4Fe-4S] cluster (B6). The FdsG subunit binds one [2Fe-2S] cluster (G7) and is similar to Nqo2 of *Tt*RC I. The subunit organisation of the *Rc*FDH diaphorase unit hence matches that of *Tt*RC I including a cluster equivalent to N1a (Fig. 2b). This cluster was suggested to minimise reactive oxygen species (ROS) production due to flavin semiquinone radicals in the presence of oxygen by temporarily storing electrons^26^, inducing structural re-arrangements that stabilise NAD^+^ binding upon reduction^28^, or increasing overall enzyme stability^29^. Although the diaphorase structure of *Rc*FDH shares high similarity with those of other oxidoreductases, the amino-terminal ferredoxin-like domain of FdsB is absent in related NADH-quinone oxidoreductases and the NAD^+^-reducing [NiFe] hydrogenases (Supplementary Fig. 3).

FdsD has been shown to positively influence the insertion of bis-MGD into *Rc*FDH^6^ and was predicted to either function as a chaperone for bis-MGD insertion or to stabilise the quaternary structure of FdsA. Homologues of FdsD can only be found in NAD^+^-dependent formate dehydrogenases, but not in other formate dehydrogenases or formylmethanofuran dehydrogenases. Up to now, no structural data are available on FdsD. Our cryo-EM map reveals that FdsD folds into a four-helix bundle resembling domain 1 of methionine synthase^30^, albeit no apparent sequence homology to known proteins was identified. FdsD interfaces with 23 out of its 69 amino acids with both MGD coordinating domains of FdsA and with a loop that extends from the C-terminal cap domain shielding the bis-MGD cofactor (Fig. 2c). Interaction is mediated by several hydrogen bonds, by three salt bridges, and by aromatic stacking between Trp^917^ in FdsA and two conserved phenylalanines (Phe^16^, Phe^17^) in FdsD (Fig. 2c, Supplementary Fig. 4a). Trp^917^ is located at the tip of the cap loop of FdsA and is highly conserved in all FDH complexes predicted to contain FdsD (Fig. 2d). Both ends of the loop contain conserved aromatic and charged residues that interact with both MGD cofactors of FdsA. We hypothesise that the cap loop functions as a sensor for bis-MGD insertion during FDH assembly. FdsD binding to FdsA locks the cap domain of FdsA in place and might prevent damage or loss of the cofactors.

### The Mo active site of *Rc*FDH

**Fig. 3:**
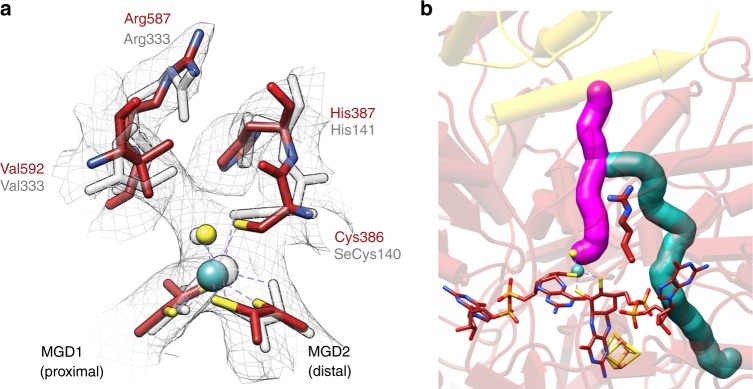
The active site of *Rc*FDH. **a** Superimposition of active site structural elements of oxidised FdhF (PDB-ID: 1fdo [10.2210/pdb1FDO/pdb], transparent white) with those of the as isolated *Rc*FDH active site (red). The map of the as isolated structure is shown as grey mesh. **b** Proposed hydrophilic (pink) and hydrophobic (cyan) tunnels in *Rc*FDH. FdsA (red) and FdsD (gold) subunits are depicted as pipes and planks with Arg587, bis-MGD and cluster A1 shown as sticks.

The bis-MGD containing active site of as isolated *Rc*FDH structurally resembles that of oxidised FdhF (Fig. 3a, Supplementary Movie 1). The EM map around molybdenum indicates six ligands coordinated in a trigonal prism geometry. The rectangular base of the prism is formed by the two dithiolene groups of the bis-MGD molecule coordinating molybdenum from one side. The two remaining coordination sites are occupied by the Cys^386^ sulphur and a small ligand that is oriented towards Val^592^. As evidenced by the full reduction of the bis-MGD containing enzyme with formate (Supplementary Fig. 1c), a terminal sulfido ligand likely occupies this site^18^. The active site residues Arg^587^ and His^387^ are proposed to position formate for C–H bond cleavage and to elevate the p*K*_*a*_ of the cysteine ligand, respectively^15^. The side chain orientation of both residues and the coordination of molybdenum largely resembles the arrangement observed in oxidised FdhF (PBD-ID 1fdo [10.2210/pdb1FDO/pdb]) indicating that the active site molybdenum of *Rc*FDH is present in the oxidised state. There is no clear evidence for stochiometric binding of azide in any particular location of the EM map. Conversely, azide inhibition of *Rc*FDH under the conditions used in cryo EM is not mediated by stochiometric, direct binding to molybdenum.

Two tunnels can be identified in *Rc*FDH starting at the molybdenum and separating at the active site residue Arg^587^ into different exits (Fig. 3b). The pore of the shorter tunnel is mainly formed by polar and charged residues suggesting channelling of hydrophilic substrate from an entry site near FdsD to the active site. The tunnel forming residues show high conservation to those in oxidised FdhF (Supplementary Table 2). The second tunnel bears predominantly hydrophobic residues suggesting the possibility of gas transport to or away from the active site. FdhF contains a similar hydrophobic channel, which is however blocked by Val^145^ and Met^157^ in place of glycine residues at these position in *Rc*FDH (Supplementary Fig. 4b, Supplementary Table 3). Albeit the path of the hydrophobic tunnel differs from the CO_2_ tunnel proposed for formyl-methanofuran dehydrogenase^3^, the active site residue Arg^587^ might analogously control gate opening to each channel facilitating efficient catalysis. Intriguingly, the glycine residues are conserved in NAD^+^-dependent FDHs while other FDHs display larger hydrophobic residues at this position effectively occluding the tunnel (Supplementary Fig. 4c).

### Cofactor arrangement in *Rc*FDH

The direct electron transfer chain between bis-MGD and FMN of the heterotetramer measures 76 Å and consists of five Fe-S clusters: A1, A2, A3, A5 and B6 (Fig. 2a). All edge-to-edge electron transfer distances account for <14 Å and are thus within a reasonable distance for physiological electron transfer^31^. The high structural similarity between *Tt*RC I and *Rc*FDH is reflected in a good match of the Fe-S cluster positioning, when the structures are superimposed (Fig. 2b). The remote cluster N7 in complex I corresponds to cluster A1 in *Rc*FDH, which can receive an electron from the reduced molybdenum atom. While cluster N7 is disconnected from other Fe-S clusters and is regarded as an evolutionary remnant in *T. thermophilus*, cluster A2 couples cluster A1 with the remaining electron transfer chain in *Rc*FDH. The existence of a [4Fe-4S] cluster in a position equivalent to A2 in respiratory complex I of *Campylobacter jejuni*, *Helicobacter pylori* and *A. aeolicus* was predicted from sequence analysis^32,33^, but the cluster has not been resolved structurally. Similarly, the position of the A2 cluster was predicted in a model of *C. necator* FDH based on structures of FdhF and *Tt*RC I^34,35^.

Two additional clusters lie outside the electron transfer pathway from bis-MGD to FMN: [2Fe-2S] cluster G7, which is in proximity to the FMN cofactor, and [4Fe-4S] cluster A4 at the *Rc*FDH dimer interface. Cluster A4 is coordinated by Cys^121^, Cys^124^, Cys^130^ and His^117^. The two Cys^121^ residues are located directly at the dimer interface and lie within good disulphide-bonding distance, but the EM map suggests they are essentially coordinated to their corresponding cluster (Supplementary Fig. 4d). The FdsA subunit interface is formed by several hydrogen bonds and hydrophobic interactions. In particular, Cys^121^ is flanked by two intercalating leucine residues (Leu^119^, Leu^122^), which are conserved in NAD-dependent FDHs, but not in complex I (Supplementary Fig. 4e). Leu^122^, which has the largest buried surface area in the interface, is more conserved than Leu^119^. The four Fe-S clusters A3-A4-A4′-A3′, lie all within a similar electron transfer distance (Fig. 2a) and potentially allow for electron transfer between the two FDH protomers. For membrane-bound [NiFe] hydrogenase I of *E. coli* it has been shown that electron transfer between protomers in the quaternary structure is important for recovery of the active site after O_2_ attack^36,37^. This mechanism contributes to the oxygen tolerance of [NiFe] hydrogenases. The concrete nature of cluster A4’s function will be the subject of future studies.

### NADH-reduced structure of *Rc*FDH

Our EPR spectroscopic characterisation shows that NADH treatment results in partial generation of the paramagnetic Mo^V^ oxidation and respective reduction of the Fe-S clusters, without generation of an FMN^•^ radical (Supplementary Fig. 6 and Supplementary Table 4). The Mo^V^ and detected Fe-S clusters bear resemblance to the FDHs characterised from *Methanobacterium formicicum* and *Methylosinus trichosporium*, and more recently from *Cupriavidus necator*^21,34,38^. Qualitatively, respective measurements show that these cofactors are partially reduced following NADH treatment (Supplementary Fig. 5a, b). The nonquantitative integrated spin concentration at 12 K (reflecting weakly power-saturated [4Fe-4S] clusters) and 80 K (reflecting slow-relaxing Fe-S clusters and Mo^V^) and the decreased spin concentration relative to the stronger reductant sodium dithionite support this assessment (Supplementary Table 4), in addition to the comparable partial reduction of FMN and Fe-S clusters by NADH observed at NADH:FDH ratios of 4000 and 20 as recorded by UV-visible spectroscopy (Supplementary Fig. 5c, d). EPR spectroscopy and UV-Vis reduction spectra collectively reflect an enzyme that has underwent incomplete reduction by NADH. This behaviour is consistent with previous reports on other molybdoenzymes like xanthine dehydrogenase^39^. The inability of NADH to completely reduce the enzyme might be dependent on the redox potentials of the cofactors. Furthermore, the presence of highly inhibitory concentrations (10 mM) of azide might prevent complete reduction of the enzyme.

In order to structurally investigate this partially reduced state of the enzyme, we determined the cryo-EM structure of *Rc*FDH in the presence of NADH and azide at 3.2 Å resolution (Supplementary Fig. 6). The atomic model derived from this structure overlays with the coordinates of the as isolated state with a root mean square deviation of 0.31 Å over all but 20 Cα atoms of the four peptide chains (Supplementary Fig. 7, Supplementary Table 1). The strong similarity between both structures extends to the active site residues and cofactors, whose location and orientation are indistinguishable between the two states (Supplementary Movie 2). None of the structures shows dissociation of Cys^386^ from the active site Mo. Also, no structural changes were observed at the pyranopterins of the bis-MGD likely reflecting that the cofactor has not been reduced to the Mo^IV^ state^40^.

**Fig. 4:**
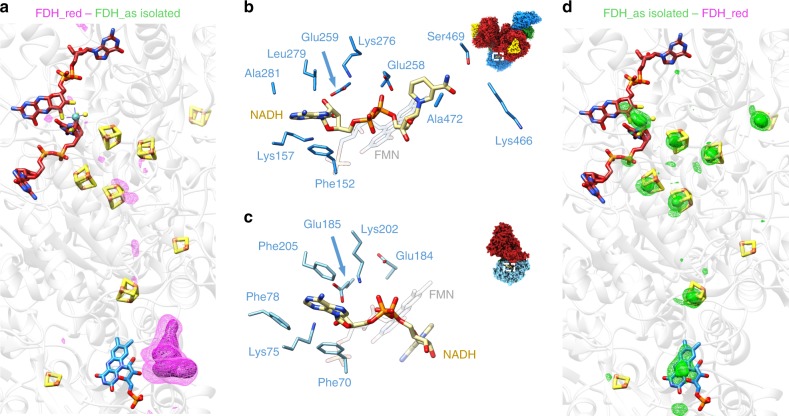
The NADH-reduced structure of *Rc*FDH. **a** Surface representation of difference map (magenta mesh at 18 σ and 11 σ) between the NADH reduced and the as isolated cryo-EM maps. The atomic model overlaid with the as isolated cryo-EM structure is shown as white ribbon with coloured cofactors. **b** Close-up of the NADH binding site in the NADH-reduced structure of *Rc*FDH. NADH interacting residues and the position of FMN are indicated **c** Close-up of the NADH binding site in *Tt*RCI (PDB-ID 3iam [10.2210/pdb3IAM/pdb]). **d** Surface representation of difference map (green mesh at 18 σ and 11 σ) between the as isolated and the NADH reduced cryo-EM maps.

The difference density between the maps of the NADH reduced and the as isolated enzyme shows a distinct density for NADH near the FMN binding site (Fig. 4a). Intriguingly, NADH binding to FdsB differs from that to the homologous diaphorase unit of *Tt*RC I (PDB-ID 3iam [10.2210/pdb3IAM/pdb]). Instead of stacking underneath the isoalloxazine ring of FMN, the nicotinamide moiety of NADH sits in front of the FMN binding pocket in hydrogen bond distance to the backbone of FdsB (Fig. 4b, c, Supplementary Movie 3). In this position it blocks access to the binding pocket and also prevents FMN from disengaging from the enzyme, but it is too far away (11.6 Å) to allow for productive electron transfer between NADH and FMN. In contrast, the coordination of the adenosine diphosphate (ADP) moiety of NADH resembles that of complex I. Conserved residues Glu^259^ and Lys^157^ form hydrogen bonds to the ribose oxygens of NADH and the alcohol groups of FMN contact the second phosphate group of ADP (Fig. 4b). Furthermore, the adenine stacks against Phe^152^ of FdsB in analogy to Phe^70^ of Nqo1 in *Tt*RC I. Upon NADH binding to complex I, a hydrogen bond between Lys^202^ and Glu^184^ is broken and Lys^202^ forms a bond to NADH^41^. Despite being conserved in this position, the homologous residue Lys^276^ of FdsB does not engage with NADH or relocate between the two examined states. More importantly, the negative charge on Glu^258^ in FdsB repels the phosphate residues of NADH and may contribute to the non-productive positioning of the nicotinamide moiety. We have already shown that NADH can be used as an electron donor for the reduction of CO_2_ to formate^6^. It is hence expected that NADH can bind the diaphorase unit of *Rc*FDH productively to deliver electrons to the electron transfer chain via FMN. At 3.2 Å resolution we are unable to distinguish NADH from NAD^+^, but considering the estimated 100-fold excess of NADH over NAD^+^ at the time of grid preparation and the fact that none of the residues in proximity of the nicotinamide nitrogen atom are suitable to stabilise a positive charge of NAD^+^, we have likely trapped NADH bound to the substrate inhibited enzyme in our cryo-EM structure.

### Reduction of the *Rc*FDH cofactors

When subtracting the map of the NADH reduced enzyme from that of the as isolated enzyme, the difference map essentially shows densities at all cofactors of the electron transfer chain (Fig. 4d). We conclude that the density in these regions of the as isolated EM structure are stronger than in the map of the NADH bound enzyme. This effect can either arise if the cofactors in the NADH bound enzyme move or if they show a different scattering behaviour due to charge. High-resolution crystallographic characterisation of the high-potential iron–sulfur protein in different redox states, indicate a small (up to 0.03 Å) contraction of the oxidised [4Fe-4S] cluster^42^ with regard to the reduced cluster. These movements are too small to be visualised in our 3D reconstructions and would also not explain the observed densities around molybdenum, phosphates or FMN. EM maps reflect the charge of atoms^43^, as scattering of electrons by atoms in electron microscopy produces coulomb potential maps. In particular at resolution ranges between 5 and 10 Å, atomic scattering amplitudes are usually weaker the more negatively charged atoms are^44^. Hence, the difference map between the unsharpened EM maps of the as isolated and the NADH reduced states of FDH qualitatively visualises negative charges on electron accepting atoms. When the maps are B-factor sharpened to weigh down low resolution frequencies and boost high-resolution frequencies, the signal in the difference maps disappears, which is in agreement with the observation that scattering amplitudes differ very little at high-resolution ranges (Supplementary Fig. 8). As observed in our EPR studies, NADH treatment generates Mo^V^ states in a fraction of the sample, requiring electrons to travel along the entire electron transport pathway from FMN to Mo. The difference map provides a snapshot of all electron positions as an average charge change over all complexes in the EM analysis. Weak difference densities at the [2Fe-2S] clusters could arise due to fast electron transfer or reduction of these clusters in both states, though EPR spectroscopic characterization of the as-isolated state showed no reduction of Fe-S clusters. However, it cannot be ruled out that the [2Fe-2S] clusters may be partially reoxidised after NADH treatment, e.g. because they could be more prone to autooxidation during grid preparation. The difference density at [4Fe-4S] cluster A4 indicates that this cluster is likely redox active upon NADH reduction.

## Discussion

We report the cryo-EM structure of the molybdoenzyme *Rc*FDH. It reveals an unexpected subunit composition as a dimer of FdsABGD heterotetramers. The arrangement of Fe-S clusters resembles that of complex I, supporting the idea that complex I and NAD^+^-dependent FDH evolved from the same ancestor^45^. Intriguingly, *Rc*FDH can be loaded with electrons from the FMN binding site in the presence of NADH. The lack of structural changes at the bis-MGD pterin and dithiolenes indicates that redox changes of Mo^VI^ to Mo^V^ by NADH appear to principally involve the Mo metal ion. Our cryo-EM analysis of two different redox states of *Rc*FDH shows that NADH reduction leads to charging of the cofactors in the absence of the second substrate at the bis-MGD. Since the experimentally obtained 3D density reflects the coulomb potential, our study proves that cryo-EM can indeed serve as a powerful tool to visualise charges on the cofactors of redox proteins, either by direct comparison of distinct redox states or by comparison of electron density maps with coulomb potential maps.

## Methods

### Cloning, protein expression and purification

The *fdsGBACD* operon was amplified using primers listed in Supplementary Table 6. The resulting fragment was cloned downstream of the *nifH* promotor with an N-terminal His_6_-tag before *fdsG* into vector pBBR1-MCS2^46,47^ creating the plasmid designated pTHfds36. Protein expression was performed under anaerobic (photoheterotrophic) conditions in RCV medium^48^ in *R. capsulatus* strain 37b4 supplemented with 150 µM molybdate, 9.5 mM l-serine, 25 mM DMSO and 25 µg/mL kanamycin. Main cultures were grown for 48 h at 30°C. Harvested cells were resuspended in lysis buffer (50 mM phosphate, 300 mM NaCl, 10 mM imidazole, 10 mM NaN_3_, pH 8.0) and lysed by two passages through a homogeniser (Constant Systems Ltd) at 1.35 kbar. The clarified supernatant was applied to a gravity-fed Protino® Ni-NTA (Macherey-Nagel) column. Flow through was reapplied once and the column was washed with 20 bed volumes of lysis buffer followed by 20 bed volumes of wash buffer (50 mM phosphate, 300 mM NaCl, 20 mM imidazole, 10 mM NaN_3_, pH 8.0). *Rc*FDH was eluted in elution buffer (50 mM phosphate, 300 mM NaCl, 250 mM imidazole, 10 mM NaN_3_, pH 8.0). Pure *Rc*FDH was acquired by subsequent size exclusion chromatography using a HiLoad 16/600 Superdex 200 pg column (GE Healthcare) with 75 mM K_2_HPO_4_, 10 mM NaN_3_, pH 7.5 as running buffer. The *k*_*cat*_ of the enzyme for formate oxidation was determined as 3583 ± 115 min^−1^.

### Determination of cofactor saturation

Molybdenum and iron content were determined by Inductively Coupled Plasma Optical Emission Spectroscopy (ICP-OES). Briefly, 500 µL of 20 µM *Rc*FDH was wet ashed at 100 °C overnight with an equivalent volume of 65 % HNO_3_. Samples were then diluted with 4 mL of doubly distilled H_2_O and were applied to an Optima 2100 DV instrument (PerkinElmer Life Sciences, Waltham, MA). The multielement standard XVI (Merck, Darmstadt, Germany) was used for calibration and quantification. *Rc*FDH showed a Mo saturation of 46.9% ± 3.5 and a Fe saturation of 47.1% ± 0.4 (in relation to a full occupancy with 1x bis-MGD, 5x [4Fe-4S] clusters and 2x [2Fe-2S] clusters per protomer).

### UV-visible and electron paramagnetic resonance spectroscopies

UV-visible spectra obtained for *Rc*FDH were obtained either on a Shimadzu 1280 spectrophotometer housed in an anaerobic Coy chamber (Grass Lake, MI) (O_2_ < 10 ppm) or aerobically on a Shimadzu 2600 spectrophotometer. Aerobically purified enzyme was brought into the anaerobic chamber and was made anaerobic via PD-10 buffer exchange columns (GE Healthcare) into degassed 100 mM Tris-HCl, 10 mM NaN_3_, pH 9.0 and was concentrated anaerobically to ~ 500 µM using a centrifuge (1-15PK, Sigma, Germany) at 14,000×*g*. Enzyme used for aerobic experiments was prepared similarly using aerobic buffer. In all, 2–5 µM FDH was treated either with sodium formate (5 mM final concentration) or NADH (40 µM or 20 mM final concentration). Following this, the above sample was treated with sodium dithionite (2 mM final concentration).

EPR samples were prepared aerobically in an ice bath using FDH purified as above, but afterward was desalted with a PD-10 column into in 100 mM Tris-HCl, 10 mM NaN_3_, pH 9.0. Typical EPR sample preparation methods involved addition of 20 µl of the above buffer or either freshly-prepared 100 mM NADH or sodium dithionite to 180 µl of as-isolated *Rc*FDH residing in a quartz EPR capillary (3.9 mm O.D.), followed by brief mixing and relatively immediate freezing (10–15 s) in a liquid N_2_-cooled ethanol bath before final freezing in liquid N_2_. The final concentration of NADH or dithionite was 10 mM.

CW X-band EPR spectra were obtained using a laboratory-built spectrometer (microwave bridge, ER041MR, Bruker; lock-in amplifier, SR810, Stanford Research Systems; microwave counter, 53181 A, Agilent Technologies) equipped with a Bruker SHQ resonator. An ESR 910 helium flow cryostat with an ITC503 temperature controller (Oxford Instruments) was used for temperature control. A Cu(II)/EDTA standard was used as a reference for spin quantitation of FDH samples^49^. Spin quantitation (by double integration) was performed using the utility ‘spincounting’ (https://github.com/lcts/spincounting) in Matlab (Mathworks). Magnetic field calibrations were applied through measuring a reference N@C_60_ sample (*g* = 2.00204) at ambient temperature to compensate field offsets between Hall probe and sample position^50,51^. Parameters for EPR data acquisition at 12 K were: modulation amplitude, 5 G; microwave power, 4.0 mW; microwave frequency, 9.38 GHz. Parameters for EPR data acquisition at 80 K were identical, except that the modulation amplitude was 2 G.

### Electron microscopy data acquisition

In all, 3.5 µl of as isolated *Rc*FDH (100 µg/ml) was either directly applied to freshly glow-discharged Quantifoil R2/4 300-mesh holey carbon grids with 2 nm carbon support films or after 5 min incubation with 2 mM NADH. The protein solution was incubated on the grid for 45 s at 5 °C and 85% humidity before blotting for 2 s and plunge freezing in liquid ethane using a FEI Vitrobot.

Cryo-EM images for the initial model were collected under low-dose conditions on a FEI Spirit microscope operated at 120 kV equipped with a 4kx4k F416 CMOS camera (TVIPS). We used CTFFIND4^52^ for estimation of the contrast transfer function parameters, and Relion 1.4^53^ or Imagic^54^ for all subsequent steps. 1014 manually selected particles were subjected to 2D classification in Relion 1.4 in order to obtain references for template-based particle picking. Semi-automated particle selection, z-score sorting and 2D classification in Relion resulted in a FDH dataset of 116783 particles. Particle images were normalised, band-pass filtered between 200 and 10 Å, and classified using multivariate statistical analysis in IMAGIC. The class averages were used to generate an initial 3D reconstruction by angular reconstruction imposing two-fold symmetry. This reconstruction was submitted to 3D auto-refinement in Relion 1.4 resulting in a map with a final resolution of 14.13 Å.

High-resolution cryo-EM images of as isolated *Rc*FDH were collected on a FEI Tecnai G2 Polara microscope operated at 300 kV equipped with a Gatan K2 Summit direct electron detector. 4623 micrographs were recorded in super-resolution mode at a pixel size of 0.628 Å using LEGINON^55^. The defocus range was set from −0.6 to −3.2 µm. Each micrograph was dose-fractionated to 50 frames with a total exposure time of 10 s and a total dose of 64 e^−^/Å^2^. The first 25 frames were used for image processing.

High-resolution cryo-EM images of NADH incubated *Rc*FDH sample were collected on a FEI Tecnai G2 Polara microscope operated at 300 kV equipped with a Gatan K2 Summit direct electron detector and on a FEI Titan Krios microscope operated at 300 kV equipped with a Gatan K2 Summit direct electron detector. On the Polara microscope, 4082 micrographs were recorded in super-resolution mode at a pixel size of 0.628 Å using LEGINON. The defocus range was set from −0.6 to −3.5 µm. Each micrograph was dose-fractionated to 50 frames with a total exposure time of 10 s and a total dose of 64 e^−^/Å^2^. On the Krios microscope, 3984 micrographs were recorded in counting mode at a pixel size 1.1 Å. The defocus range was set from −0.6 to −3.2 µm. Each micrograph was dose-fractionated to 50 frames with a total exposure time of 8 s and a total dose of 40 e^−^/Å^2^. The first 25 frames were used for image processing.

### Cryo-EM image analysis

Image processing and 3D reconstruction was performed using RELION-3.0^56^. Movie frame alignment and dose-weighting was performed with MotionCor2^57^ and contrast transfer functions were determined using CTFFIND4. All refinements used gold standard Fourier shell correlation (FSC) calculations and reported resolutions are based on the FSC = 0.143 criterion of mask corrected FSC curves. All maps were masked and sharpened using automatically determined negative B-factors. Supplementary Figures 2 and 6 as well as Supplementary Table 5 show all steps of image processing of each of the datasets. Channel cavities were detected using the CAVER 3.0.1^58^ plugin for PyMOL (The PyMOL Molecular Graphics System, Version 2.0 Schrödinger, LLC.) with following settings: probe radius, 0.97; shell radius, 3.0; shell depth, 8.0; frame weighting coefficient, 1.0; frame clustering threshold, 1.0. Micrographs of as isolated *Rc*FDH sample showing strong astigmatism, over focus, very low defocus, broken ice or ice contaminations were discarded, resulting in 4347 micrographs for further processing steps. 2D classification of 1237 manually picked particles generated templates for semi-automated particle selection. The dataset of 1117847 particles was subjected to z-score sorting and several iterative 2D classifications to remove bad particles, resulting in a final dataset of 799023 *Rc*FDH particles. 3D classification with exhaustive angular searches were performed to further clean the dataset resulting in one good class with 366558 particles which was used for subsequent 3D auto-refine. The initial model was filtered to 40 Å and used as a reference for 3D auto-refinement of the entire dataset resulting in a *Rc*FDH reconstruction at 3.43 Å resolution without applied symmetry and 3.30 Å resolution with applied C2 symmetry, respectively. CTF per particle Refinement of the C2 symmetry map resulted in 3.26 Å resolution and was used for model building.

All frames were used for image processing. Micrographs of NADH incubated *Rc*FDH sample showing strong astigmatism, over focus, very low defocus, broken ice or ice contaminations were discarded, resulting in 4082 micrographs of the FEI Polara dataset and 3216 micrographs of the FEI Titan Krios dataset for further processing steps. For semi-automated particle selection, the templates of the oxidised dataset were adjusted to the corresponding boxsize and pixelsize. The dataset of 1215305 (FEI Polara dataset) and 744234 (FEI Titan Krios dataset) particles was subjected to z-score sorting and several iterative 2D classifications to remove bad particles, resulting in a final dataset of 669964 *Rc*FDH particles from FEI Polara dataset and 370195 *Rc*FDH particles from FEI Titan Krios dataset. At this point the two datasets were merged resulting in a total pf 1037182 particles with a customised pixel size of 1.07 Å. To ensure the two datasets were merged properly an additional round of 2D classification was done resulting in a final dataset of 669976 *Rc*FDH particles. 3D classification with exhaustive angular searches were performed to further clean the dataset resulting in one good class with 199229 particles which was used for subsequent 3D auto-refine. The initial model was filtered to 40 Å and used as a reference for 3D auto-refinement of the entire dataset resulting in a *Rc*FDH reconstruction at 3.57 Å resolution without applied symmetry and 3.37 Å resolution with applied C2 symmetry, respectively. CTF per particle Refinement of the C2 symmetry map resulted in 3.24 Å resolution and was used for model building.

### Generation of difference maps

In contrast to scattering of X-rays by atoms, electron scattering amplitudes heavily depend on the charge of the atom at low resolution ranges^43,59–61^. When two experimental EM maps representing different redox states of the same complex are subtracted from each other, the difference map will visualise charge differences. This effect is strongest at resolution ranges between 5 and 10 Å, so that difference maps will indicate the charge better when produced from unsharpened maps than from sharpened maps. Difference maps were produced by subtracting refined maps prior to post-processing or after B-factor sharpening as indicated using the vop subtract command in Chimera with the option -minRMS to normalise the data. Only data obtained from the Tecnai G2 Polara microscope were used for the generation of difference maps.

### Molecular modelling

Both maps show clear side chain density for almost all residues allowing for model building by homology model-guided chain tracing. Model building of *Rc*FDH as isolated complex was carried out in COOT^62^ into the different refined density maps. Crystal structures of sequence homologous structures were used to guide model building for FdsA, FdsG and C-terminal part of FdsB. FdsD and the N-terminal part of FdsB were built de novo into density maps based on defined densities of bulky residues. For cofactor addition, unmodeled blobs were identified and filled with the respective ligand. The models were refined using phenix.real_space_refine implemented in PHENIX using additional geometry restraints for the fitted ligands. The data collection and model statistics are summarised in Supplementary Table 5. Our atomic model covers residues 7–955, 1–493, 2–149 and 2–70 in FdsA, FdsB, FdsG and FdsD, respectively. We thus obtained a structural model for almost the entire *Rc*FDH complex.

### Alignments

Clustal Omega^63^ was used for sequence alignment. Jalview 2.11.0^64^ was used for visualizations.

### Reporting summary

Further information on research design is available in the Nature Research Reporting Summary linked to this article.

## Supplementary information

Supplementary Information

Description of Additional Supplementary Information

Supplementary Movie 1

Supplementary Movie 2

Supplementary Movie 3

Reporting Summary

## Data Availability

The EM maps of the as isolated structure and the structure in the presence of NADH are deposited under accession codes EMD-10496 and EMD-10495, respectively. Atomic coordinates of FDH and structure factors derived from the as isolated map and the map obtained in the presence of NADH have been deposited in the Protein Data Bank under accession codes 6TGA (PDB-ID 6TGA [10.2210/pdb6TGA/pdb]) and 6TG9 (PDB-ID 6TG9 [10.2210/pdb6TG9/pdb]), respectively. Other data are available from the corresponding author upon reasonable request.
