# Supplementary material for: Cryo-EM structures reveal intricate Fe-S cluster arrangement and charging in Rhodobacter capsulatus formate dehydrogenase
Source: Nat Commun. 2020 Apr 20;11:1912. doi: 10.1038/s41467-020-15614-0 (PMC7171172; doi:10.1038/s41467-020-15614-0)
Supplement: Supplementary file 1 — Supplementary Information [file 41467_2020_15614_MOESM1_ESM.pdf]

Supplementary Information

Cryo-EM structures reveal intricate Fe-S cluster arrangement and charging in *Rhodobacter capsulatus* formate dehydrogenase

Christin Radon, Gerd Mittelstädt *et al.*

List of Content

Supplementary Figures 1-8

Supplementary Tables 1-6

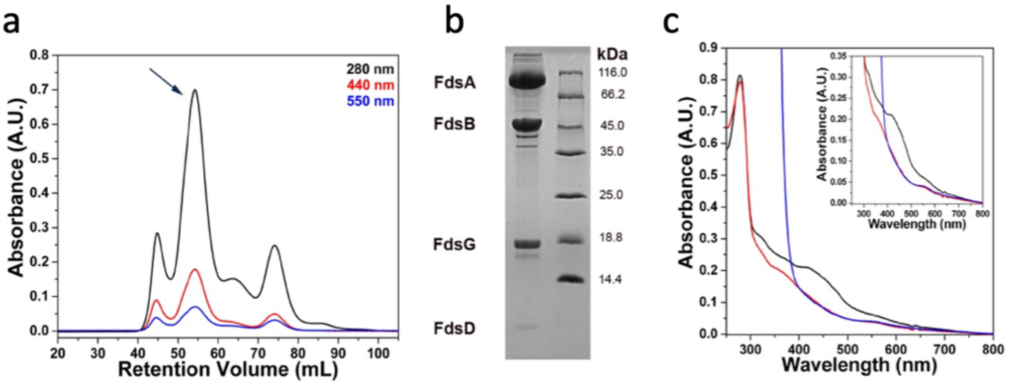

Supplementary Figure 1 | Biochemical Characterization of RcFDH. a, Size exclusion chromatography of 90 μM *Rc*FDH performed on a Superdex200pg column in 75 mM potassium phosphate, 10 mM NaN_3, pH 7.5 at 4 °C. The purified fraction corresponding to the holoenzyme is represented by the main fraction that eluted at approximately 55.0 mL and is indicated by an arrow. Elution traces represent absorbance wavelengths at 280 nm (black trace), 440 nm (red trace), and 550 nm (blue trace). **b,** 15% SDS-polyacrylamide gel of the purified *Rc*FDH holoenzyme. Gel depicts 46 µg of purified FDH. Subunits corresponding to FdsA, FdsB, FdsG, and FdsD are indicated. Reproducible gels could be obtained following at least 3 or more independent purifications. **c,** UV-visible absorption spectra of as-isolated *Rc*FDH (black trace) that was subjected to immediate reduction with 5 mM sodium formate (red trace), followed by treatment by 2 mM sodium dithionite (blue trace). Inset depicts a zoomed-in view of the features present at 250-800 nm. Spectra were recorded in 100 mM Tris-HCl, 10 mM NaN_3, pH 9.0 buffer under anaerobic conditions in a Coy chamber (< 10 ppm O_2).

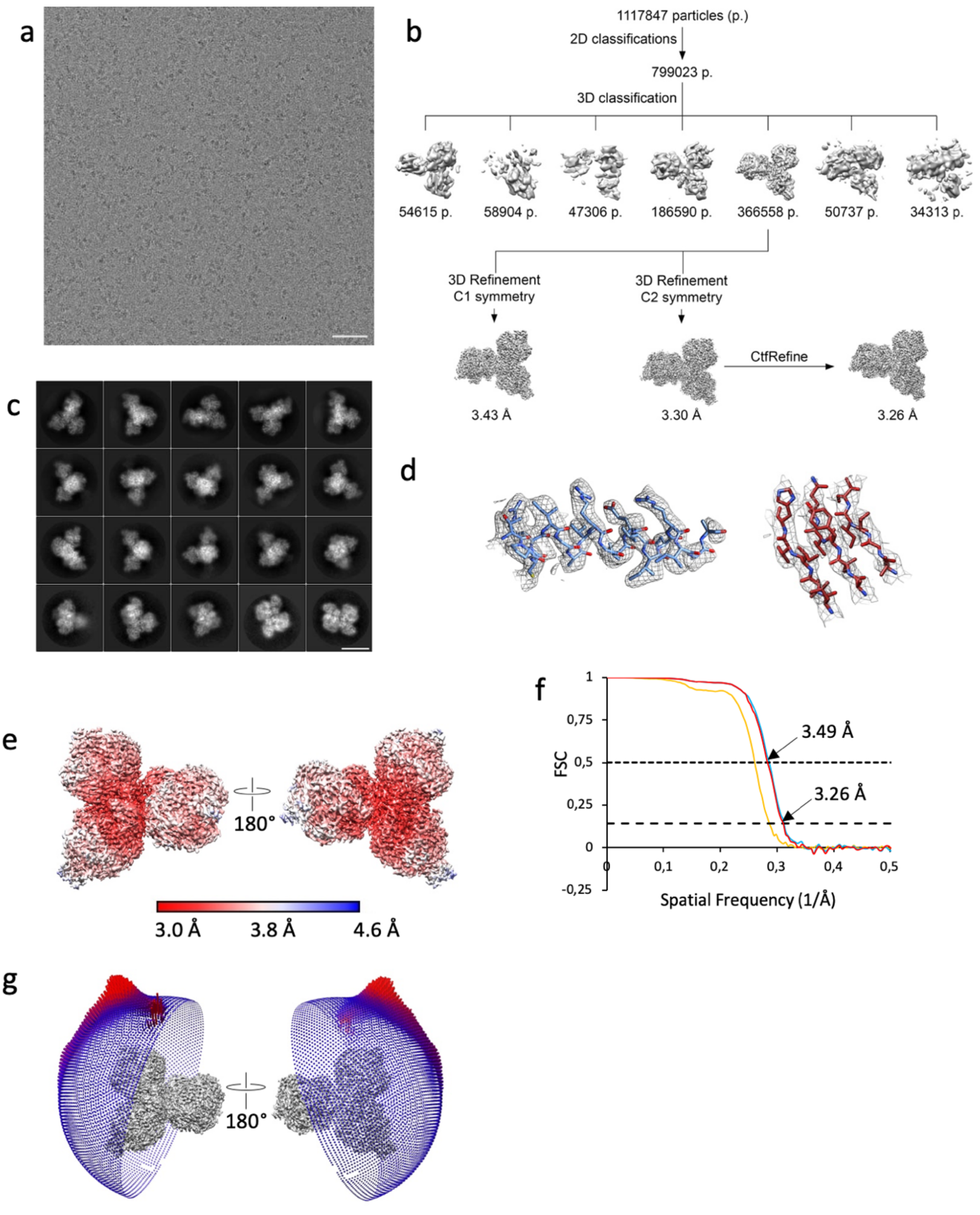

Supplementary Figure 2 | Cryo-EM analysis of *Rc*FDH. a, A representative cryo-EM micrograph of *Rc*FDH data collection. Scale bar represents 500 Å. **b,** Data processing workflow showing the image processing procedure with the number of particles and the resolution of the reconstruction for every step. **c,** Representative 2D class averages of various views of *Rc*FDH. Scale bar represents 100 Å. **d,** Mesh representation of the map showing a representative α-helix and β-sheets superimposed on the atomic model. **e,** Local resolution estimate in Relion. Side views of the density map coloured according to the local resolution estimated by Relion. **f,** Fourier shell correlation (FSC) plots for the *Rc*FDH EM map with imposed C2 symmetry. The

resolution at FSC=0.143 and FSC=0.5 are indicated. A comparison of the FSC corrected (red), masked (blue) and unmasked map (yellow) is shown. **g,** Angular distribution of the final reconstruction.

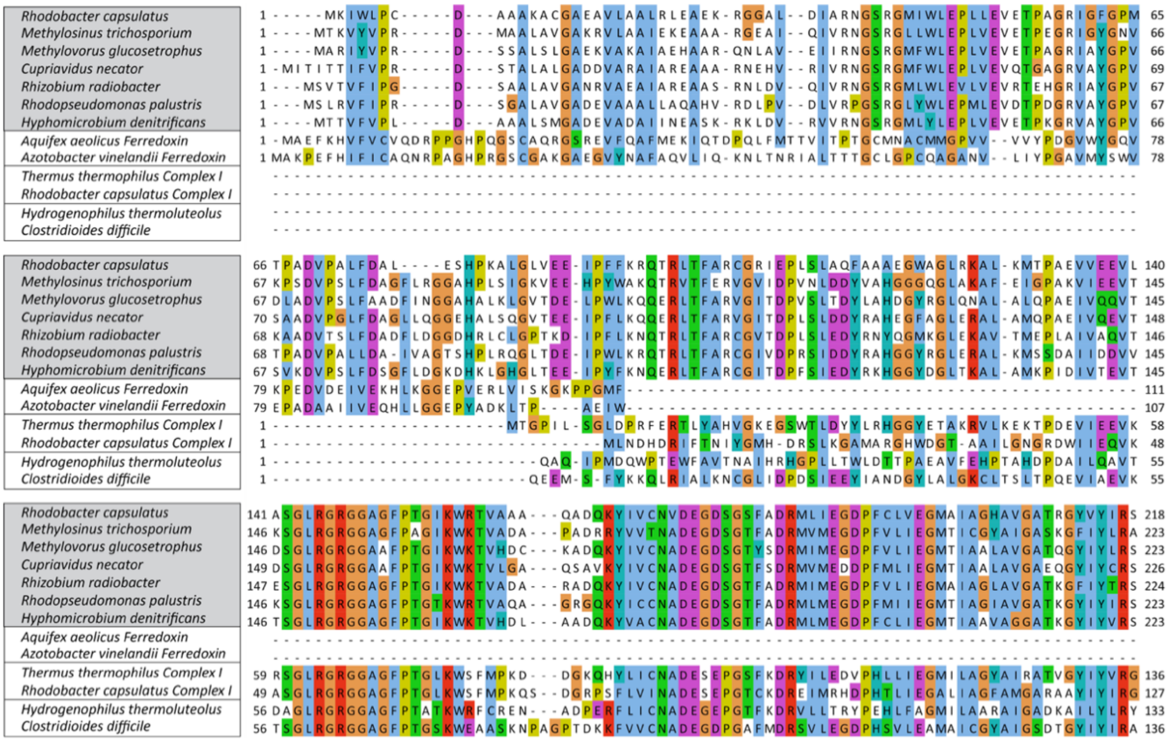

Supplementary Figure 3 | Multiple sequence alignment of FdsB homologues from NAD+-dependent FDHs, Ferredoxins, NADH-quinone oxidoreductases and Hydrogenases

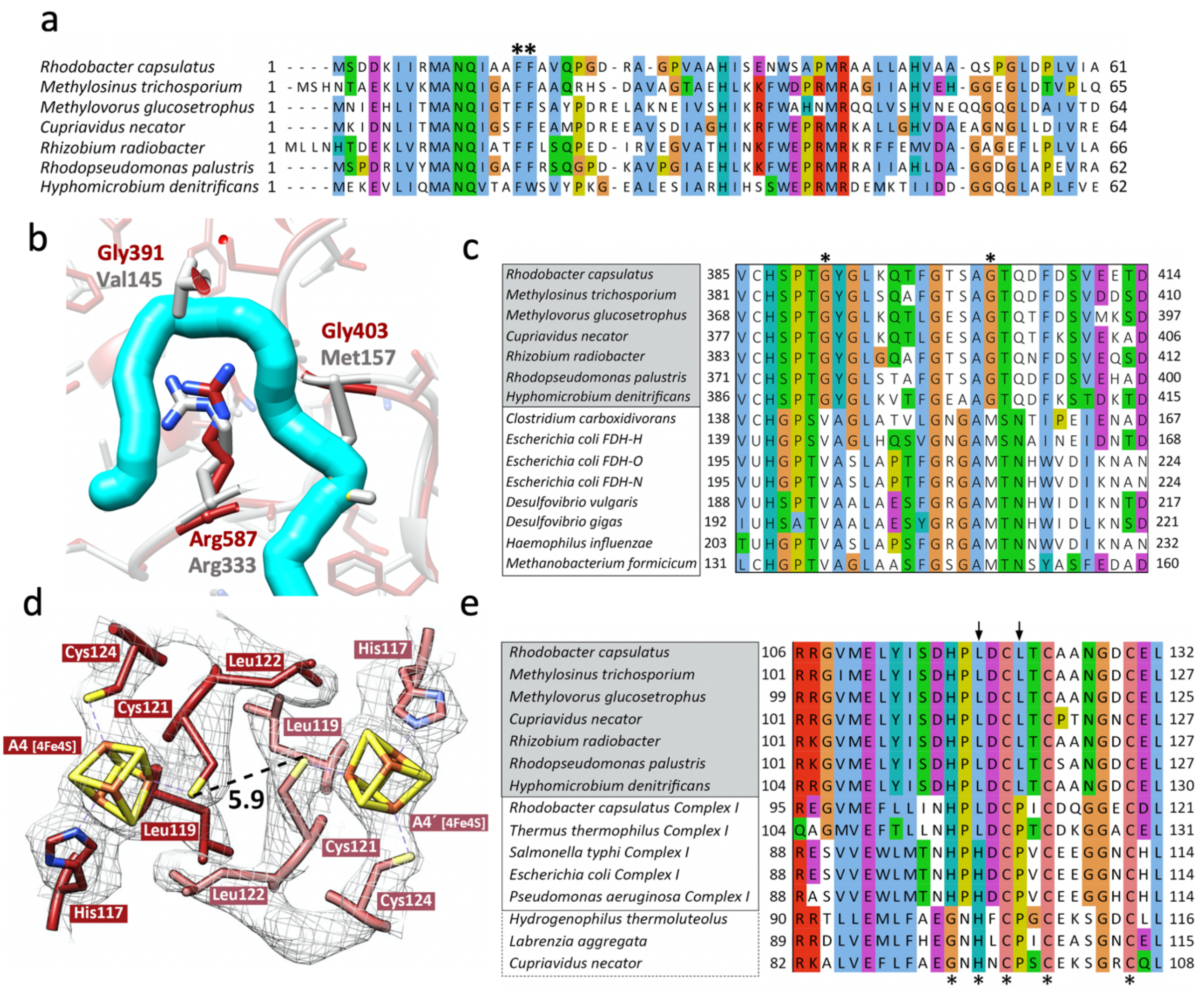

Supplementary Figure 4 | Channel and [4Fe-4S] cluster A4 of *Rc*FDH. a, Multiple sequence alignment of FdsD homologues from NAD+-dependent FDHs. Stars indicate conserved phenylalanine residues. **b,** Superimposition of *Rc*FDH (red) and oxidised FdhF (PDB-ID: 1fdo; white) structures at active site. Active site residue Arg 587 and residues Gly^{391} and Gly^{403} of RcFDH as well as Val^{141} and Met^{157} of FdhF are shown as sticks. The proposed hydrophobic channel is indicated in cyan. **c,** Multiple sequence alignment of FdsA homologues from organisms predicted to harbour FdsD (grey-shaded) or not (white-shaded). Stars indicate conserved glycine residues in NAD+ dependent FDHs which are replaced by valine and methionine, respectively, in other FDHs. **d,** Dimer interface between the two FdsA subunits. [4Fe-4S] cluster A4 and A4' are shown. Cluster coordinating residues and hydrophobic residues at the dimer interface are shown as stick representation. The distance between sulphur atoms of Cys121 is indicated in Å. **e,** Multiple sequence alignment of FdsA homologues of several FDH, complex I and NAD+-reducing [NiFe]-hydrogenase family members. Residues that coordinate cluster A4 are indicated with stars. The arrows indicate Leu^{119} and Leu^{122}.

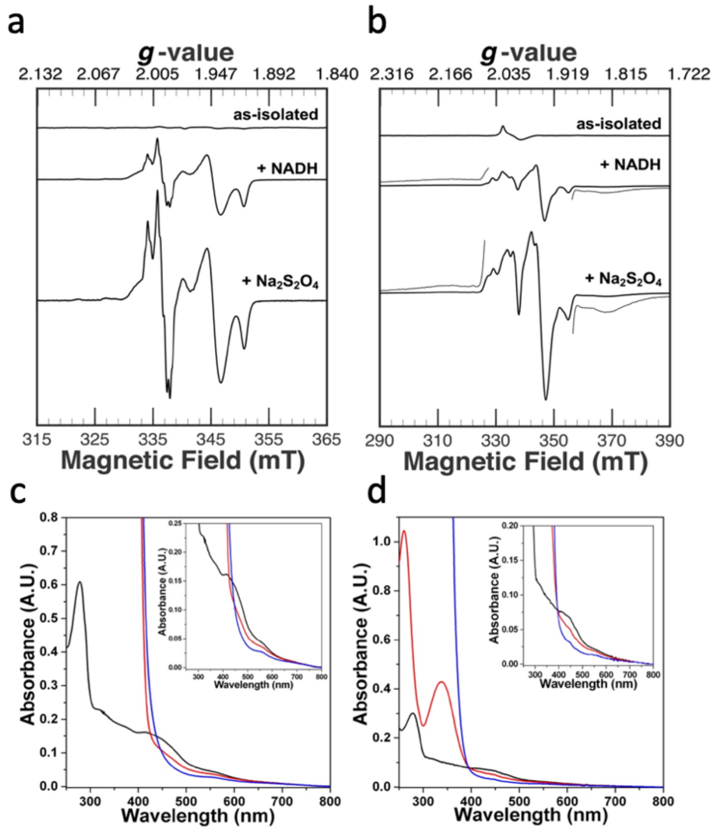

Supplementary Figure 5 | Spectroscopic characterization of *Rc*FDH. a, Constant wave X-band EPR spectra of *Rc*FDH recorded at 80 K and **b,** 12 K at a microwave frequency of 9.38 GHz. Spectra represent as isolated *Rc*FDH treated in the absence or presence of NADH or sodium dithionite. An iron-sulphur cluster signal present in the low- and high-field edges of the NADH-treated and the dithionite-treated signals is partially enlarged at a scale of 5x. **c,d,** UV-visible spectra of FDH following treatment with NADH. **a** shows the Mo^{V} and slowly-relaxing Fe-S clusters (as [2Fe-2S] or [4Fe-4S]) visible upon respective treatments. **b** emphasizes the presence of fast-relaxing Fe-S clusters that are not visible at 80 K, however some spectral components (e.g. MoV and Fe-S clusters) are power-saturated. **c** shows as-isolated FDH (3.5 μM) (black trace) that was subjected to reduction for 5 minutes at ambient temperature with 20 mM NADH (red trace), followed by treatment by 2 mM sodium dithionite (blue trace). **d** shows as-isolated FDH (1.8 μM) (black trace) that was subjected to immediate reduction at ambient temperature with 40 μM NADH (red trace), followed by treatment by 2 mM sodium dithionite. Spectra were recorded in 100 mM Tris, 10 mM NaN_3, pH 9.0 buffer under anaerobic conditions in a Coy chamber (< 10 ppm O_2). Insets depict a zoomed-in view of the features present at 280-800 nm. The reduction spectra show that the enzyme is only partially reduced by NADH, independent of the NADH concentration used and the time of reduction. The absorbance of the red trace at 340 nm in **d** shows that NADH was not completely consumed during the reduction, also visible in **d** at much higher concentrations of NADH.

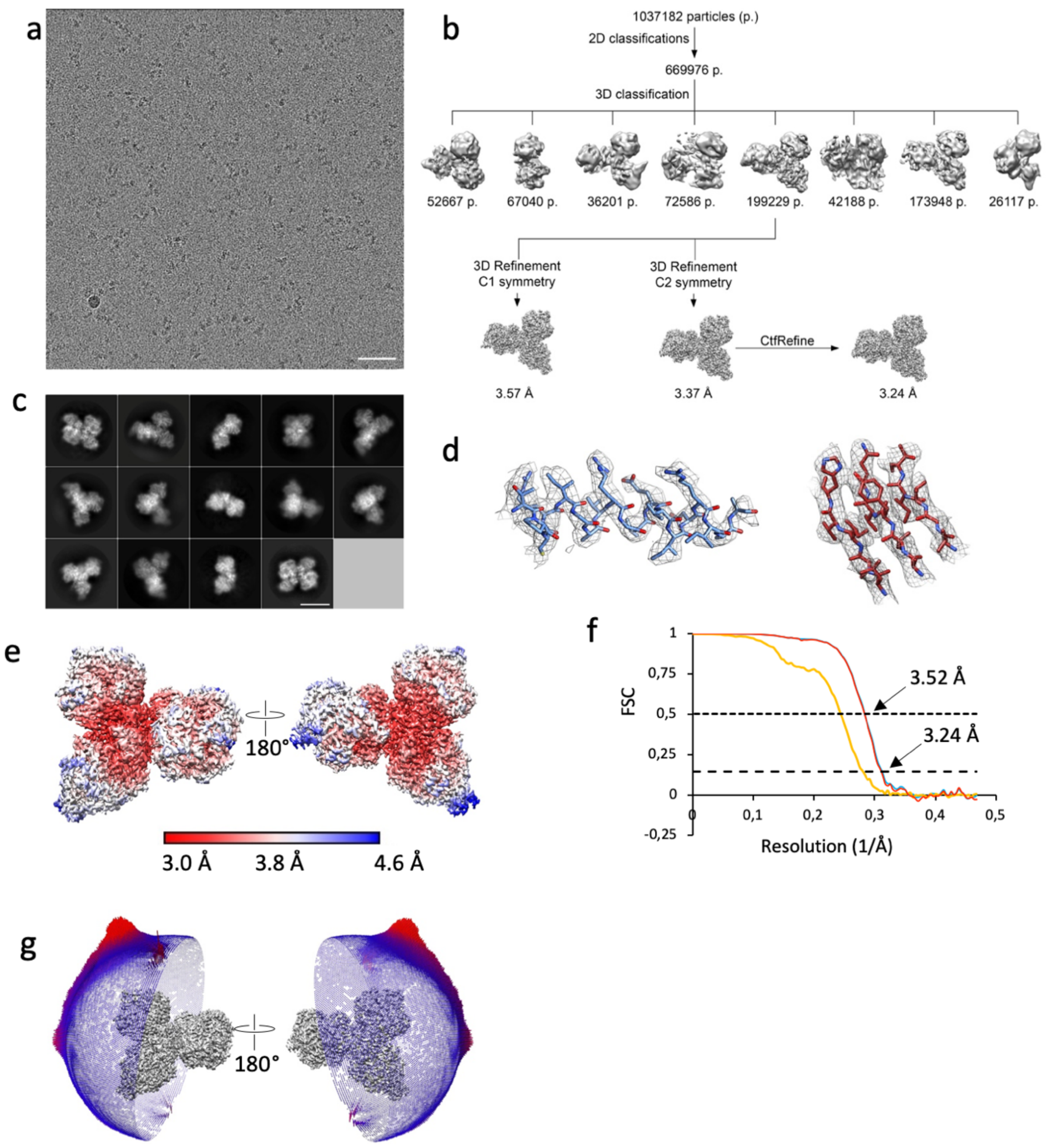

Supplementary Figure 6 | Cryo-EM analysis of NADH incubated *Rc*FDH. a, A representative cryo-EM micrograph of *Rc*FDH data collection. Scale bar represents 500 Å. **b,** Data processing workflow showing the image processing procedure with the number of particles and the resolution of the reconstruction for every step. **c,** Representative 2D class averages of various views of *Rc*FDH calculated from selected particles. Scale bar represents 100 Å. **d,** Mesh representation of the map showing a representative α-helix and β-sheets superimposed on the atomic model. **e,** Local resolution estimate in Relion. Sideview of the density map coloured according to the local resolution estimated by Relion. **f,** Fourier shell correlation (FSC) plots for the *Rc*FDH EM map with imposed C2 symmetry. The resolution at

FSC=0.143 and FSC=0.5 are indicated. A comparison of the FSC corrected (red), masked (blue) and unmasked map (yellow) is shown. **g,** Angular distribution of the final reconstruction.

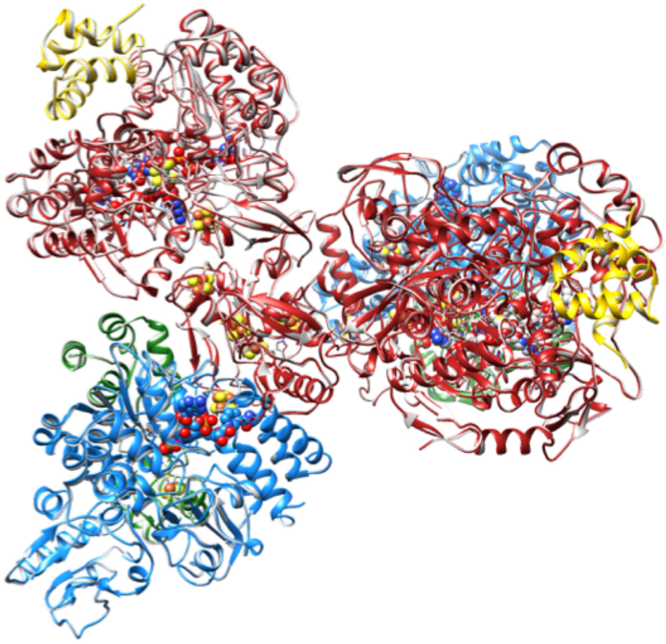

Supplementary Figure 7 | Overlay of ribbon representation of as isolated *Rc*FDH structure and NADH reduced *Rc*FDH structure. As isolated *Rc*FDH structure is shown in light grey, NADH reduced *Rc*FDH structure depicted in colour code: FdsA, red; FdsB, blue; FdsG, green; FdsD, yellow.

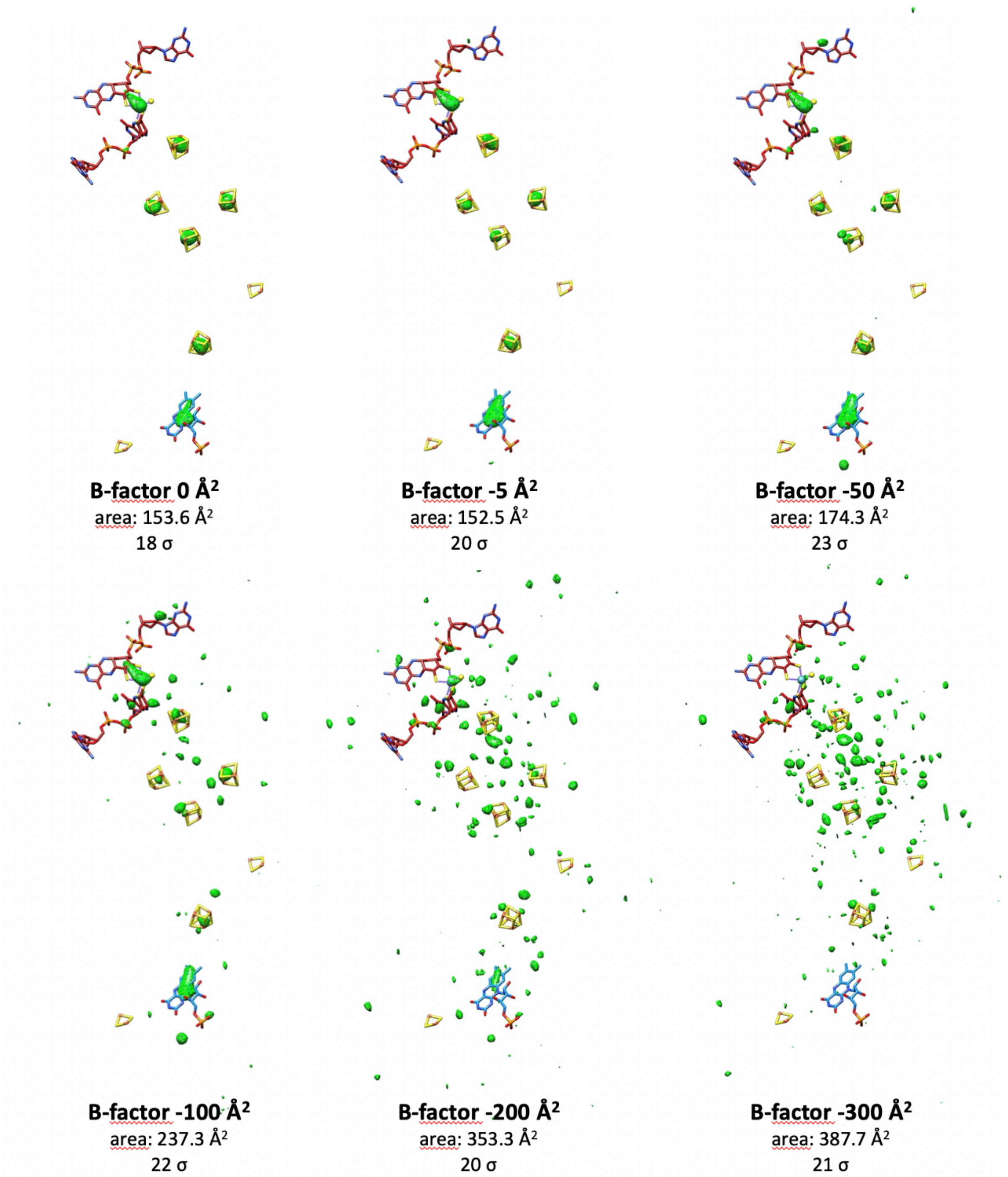

Supplementary Figure 8 | Influence of B-factor sharpening on difference maps between as isolated and NADH reduced cryo-EM maps of *Rc*FDH. Surface representations of difference maps between as isolated and NADH reduced cryo-EM maps. Cryo-EM maps of as isolated and NADH reduced FDH were B-factor sharpened prior subtraction. For comparison all difference maps were scaled to the same enclosed volume (58.8 Å^3). Applied B-factors, the total surface area and sigma values are given for each difference map. Cofactors are shown in stick representation.

Supplementary Tables

Supplementary Table 1 | Similarity scores (RMSD) of FDH components to closely related structures

RcFDH-chain[a]	pdb ID	Protein name	Homologous protein	r.m.s.d. in Å[b]	identity[c]	similarity[c]
FdsA	5xf9_B	HoxU	NAD^+-reducing [NiFe]-hydrogenase	1.159 (141)	35%	50%
FdsA	1fdo	FdhF	Formate dehydrogenase H	1.132 (554)	36%	53%
FdsA	3ml1	NapA	Periplasmatic nitrate reductase	1.110 (463)	23%	38%
FdsA	1h0h	FdhA	Tungsten containing formate dehydrogenase	1.184 (398)	21%	38%
FdsA	1kqf	FdnG	Formate dehydrogenase N	1.080 (436)	22%	36%
FdsA	3iam_3	Nqo3	Respiratory complex I	1.195 (305)	29%	44%
FdsB	1m2d	Fdx4	Thioredoxin-like ferredoxin	0.884 (31)	n.d.	n.d.
FdsB	3iam_1	Nqo1	Respiratory complex I	1.099 (311)	35%	48%
FdsG	3iam_2	Nqo2	Respiratory complex I	1.254 (88)	31%	51%
FdsABGD		FdsABGD	Formate dehydrogenase NADH-reduced	0.305 (1659)	100%	100%

[a] as isolated

[b] number of pruned Cα atoms in brackets

[c] obtained using BLAST 2 sequences

n.d. - not detected

Supplementary Table 2 | Amino acid composition of hydrophilic tunnel in *Rc*FDH and *E. coli* FdhF (PDB-ID: 1fdo)

	**RcFDH**	**1fdo**
conserved residues	FdsA-Ser^{356}	Ser^{109}
	FdsA-Cys^{382}	Cys^{136}
	FdsA-Arg^{384}	Arg^{138}
	FdsA-His^{387}	His^{141}
	FdsA-Arg^{587}	Arg^{333}
	FdsA-Gln^{589}	Gln^{335}
	FdsA-Gln^{593}	Gln^{339}
	FdsA-Cys^{596}	Cys^{342}
	FdsA-Tyr^{608}	Tyr^{356}
	FdsA-Arg^{635}	Arg^{382}
	FdsA-Thr^{919}	Thr^{673}
	FdsA-Lys^{395}	His^{149}
	FdsA-His^{603}	Asp^{349}
	FdsA-Glu^{604}	Thr^{350}
exclusive	FdsA-Ser^{388}	
	FdsA-Ser^{401}	
	FdsD-Asn^{33}	
		Cys^{135}
		Asp^{134}
		Tyr^{586}

Supplementary Table 3 | Amino acid composition of hydrophobic tunnel in *Rc*FDH and *E. coli* FdhF

	***Rc*FDH**	**1fdo**
identical	Ala^{383}	Ala^{137}
	Leu^{394}	Leu^{148}
	Val^{592}	Val^{338}
	Pro^{585}	Pro^{331}
Conserved hydrophobic	Gly^{391}	Val^{145}
	Gly^{403}	Met^{157}*
	Phe^{407}	Ile^{162}
	Val^{429}	Ile^{183}
	Phe^{430}	Val^{184}
	Leu^{586}	Val^{332}
	Phe^{601}	Leu^{347}

*extend into tunnel

Supplementary Table 4 | *Rc*FDH spin concentration from EPR spectra

**Sample Preparation Condition**	**[Spin] (μM)**	**$[Spin]/[FDH]_{protomer}$**
as-isolated (12 K)	3	0.03
+ NADH (12 K)	38	0.38
+ NADH (80 K)	51	0.51
+ $Na_2S_2O_4$ (12 K)	131	1.31
+ $Na_2S_2O_4$ (80 K)	112	1.12

Supplementary Table 5 | EM data collection, processing and refinement statistics

	FDH as isolated (EMDB-10496) (PDB 6TGA)	FDH NADH reduced (EMDB-10495) (PDB 6TG9)
**Data collection and processing**		
Magnification	31000	31000
Voltage (kV)	300	300
Electron exposure (e–/Å^2)	64	40
Defocus range (µm)	0.5-3.2	0.6-4.4
Pixel size (Å)	0.628	1.07
Symmetry imposed	C2	C2
Initial particle images (no.)	799,023	669,976
Final particle images (no.)	366,573	199,229
Map resolution (Å)	3.26	3.23
FSC threshold	0.143	0.143
Map resolution range (Å)	3.0-4.0	3.0-4.6
**Refinement**		
Initial model used (PDB code)	*de novo*	*de novo*
Model resolution (Å)	3.26	3.24
FSC threshold	0.143	0.143
Model resolution range (Å)	3.0-4.0	3.0-4.6
Map sharpening *B* factor (Å^2)	167	130
Model composition		
Non-hydrogen atoms	25,324	25,412
Protein residues	3,318	3,318
Ligands	24	26
B factors (Å^2)		
Protein	11.78	19.72
Ligand	9.78	15.60
R.m.s. deviations		
Bond lengths (Å)	0.0007	0.014
Bond angles (°)	0.996	1.161
Validation		
MolProbity score	1.84	2.06
Clashscore	8.35	11.37
Poor rotamers (%)	0.98	0.47
Ramachandran plot		
Favored (%)	94.25	91.97
Allowed (%)	5.45	7.78
Disallowed (%)	0.30	0.24

Supplementary Table 6 | Primer sequences used in this study

	Primers (5´-3´)	
**Construct**	**His6_Csp6I_fw**	**fdsDXho_rv**
pTHfds36	CAGTACATGGGCAGCAGCCATC	GCTCGAGTGGAAACGATGAC
