# Supplementary material for: Cryo-EM structures reveal intricate Fe-S cluster arrangement and charging in Rhodobacter capsulatus formate dehydrogenase
Source: Nat Commun. 2020 Apr 20;11:1912. doi: 10.1038/s41467-020-15614-0 (PMC7171172; doi:10.1038/s41467-020-15614-0)
Supplement: Supplementary file 2 — Description of Additional Supplementary Information [file 41467_2020_15614_MOESM2_ESM.pdf]

Description of Additional Supplementary Files

File Name: Supplementary Movie 1
Description: Superimposition of bis-MDG cofactor and active site residues of as isolated RcFDH (dark red) FdhF (PDB-ID 1fdo).

File Name: Supplementary Movie 2
Description: Superimposition of bis-MDG cofactor and active site residues of as isolated RcFDH (dark red) and NADH-reduced RcFDH (magenta).

File Name: Supplementary Movie 3
Description: Comparison of FMN and NADH binding-sites in NADH reduced RcFDH and NADH bound TtRC I (PDB-ID 3iam). The NADH reduced RcFDH structure is shown as pipes and planks and coloured coded as in Figure 1. NADH interacting residues are shown as sticks and coloured in light blue in the NADH reduced RcFDH structure and white in the NADH bound TtRC I structure. Heteroatoms are shown. FMN and NADH are shown as sticks and colour coded by element.
