# Supplementary material for: Cryo-EM structures reveal intricate Fe-S cluster arrangement and charging in Rhodobacter capsulatus formate dehydrogenase
Source: Nat Commun. 2020 Apr 20;11:1912. doi: 10.1038/s41467-020-15614-0 (PMC7171172; doi:10.1038/s41467-020-15614-0)
Supplement: Supplementary file 6 — Reporting Summary [file 41467_2020_15614_MOESM6_ESM.pdf]

nature research

Corresponding author(s): Wendler, Petra

Last updated by author(s): Mar 16, 2020

# Reporting Summary

Nature Research wishes to improve the reproducibility of the work that we publish. This form provides structure for consistency and transparency in reporting. For further information on Nature Research policies, see Authors & Referees and the Editorial Policy Checklist .

## Statistics

For all statistical analyses, confirm that the following items are present in the figure legend, table legend, main text, or Methods section.

| n/a | Confirmed | |
|---|---|---|
| ☐ | ☒ | The exact sample size (*n*) for each experimental group/condition, given as a discrete number and unit of measurement |
| ☐ | ☒ | A statement on whether measurements were taken from distinct samples or whether the same sample was measured repeatedly |
| ☒ | ☐ | The statistical test(s) used AND whether they are one- or two-sided<br>*Only common tests should be described solely by name; describe more complex techniques in the Methods section.* |
| ☒ | ☐ | A description of all covariates tested |
| ☒ | ☐ | A description of any assumptions or corrections, such as tests of normality and adjustment for multiple comparisons |
| ☒ | ☐ | A full description of the statistical parameters including central tendency (e.g. means) or other basic estimates (e.g. regression coefficient) AND variation (e.g. standard deviation) or associated estimates of uncertainty (e.g. confidence intervals) |
| ☒ | ☐ | For null hypothesis testing, the test statistic (e.g. *F*, *t*, *r*) with confidence intervals, effect sizes, degrees of freedom and *P* value noted<br>*Give P values as exact values whenever suitable.* |
| ☒ | ☐ | For Bayesian analysis, information on the choice of priors and Markov chain Monte Carlo settings |
| ☒ | ☐ | For hierarchical and complex designs, identification of the appropriate level for tests and full reporting of outcomes |
| ☒ | ☐ | Estimates of effect sizes (e.g. Cohen's *d*, Pearson's *r*), indicating how they were calculated |

*Our web collection on statistics for biologists contains articles on many of the points above.*

## Software and code

Policy information about availability of computer code

| | |
|---|---|
| Data collection | Leginon (Suloway et al., 2005) |
| Data analysis | Matlab (Mathworks), CTFFind4, Relion 1.4 and 3.0, MotionCor2, Imagic5 20101013E, CAVER 3.0.1, COOT 0.8 and 0.9, Phenix 1.15.2-3472, Jalview 2.11.0, Clustal Omega (web), UCSF Chimera 1.13.1, PyMOL 2.2.2 |

For manuscripts utilizing custom algorithms or software that are central to the research but not yet described in published literature, software must be made available to editors/reviewers. We strongly encourage code deposition in a community repository (e.g. GitHub). See the Nature Research guidelines for submitting code & software for further information.

## Data

Policy information about availability of data

All manuscripts must include a data availability statement. This statement should provide the following information, where applicable:

- Accession codes, unique identifiers, or web links for publicly available datasets
- A list of figures that have associated raw data
- A description of any restrictions on data availability

The EM maps of the as isolated structure and the structure in the presence of NADH are deposited under accession codes EMD-10496 and EMD-10495, respectively. Atomic coordinates of FDH and structure factors derived from the as isolated map and the map obtained in the presence of NADH have been deposited in the Protein Data Bank under accession codes 6TGA and 6TG9, respectively.
PDB structures 3IAM and 1FDO are accessible under protein databank codes 3IAM and 1FDO.

# Field-specific reporting

Please select the one below that is the best fit for your research. If you are not sure, read the appropriate sections before making your selection.

- [x] Life sciences
- [ ] Behavioural & social sciences
- [ ] Ecological, evolutionary & environmental sciences

For a reference copy of the document with all sections, see nature.com/documents/nr-reporting-summary-flat.pdf

# Life sciences study design

All studies must disclose on these points even when the disclosure is negative.

| | |
|---|---|
| Sample size | Sample sizes of cryo EM work are given in Supplementary Table 5, Supplementary Figure 2 and 6. All intact particles were considered and the sample size was sufficient to obtain high resolution. |
| Data exclusions | Particles were excluded during classification steps based on 2d and 3D analysis. Initial and final particle numbers are given in Supplementary Table 5. Only data with broken particle complexes or random noise were excluded. |
| Replication | Cryo EM data collection was performed in duplicate. |
| Randomization | Random seed was generated with standard settings in Relion. |
| Blinding | N/A |

# Reporting for specific materials, systems and methods

We require information from authors about some types of materials, experimental systems and methods used in many studies. Here, indicate whether each material, system or method listed is relevant to your study. If you are not sure if a list item applies to your research, read the appropriate section before selecting a response.

## Materials & experimental systems

| n/a | Involved in the study | |
|---|---|---|
| [x] | [ ] | Antibodies |
| [x] | [ ] | Eukaryotic cell lines |
| [x] | [ ] | Palaeontology |
| [x] | [ ] | Animals and other organisms |
| [x] | [ ] | Human research participants |
| [x] | [ ] | Clinical data |

## Methods

| n/a | Involved in the study | |
|---|---|---|
| [x] | [ ] | ChIP-seq |
| [x] | [ ] | Flow cytometry |
| [x] | [ ] | MRI-based neuroimaging |
